# Introgression of a rare haplotype from Southeastern Africa to breed California blackeyes with larger seeds

**DOI:** 10.3389/fpls.2015.00126

**Published:** 2015-03-09

**Authors:** Mitchell R. Lucas, Bao-Lam Huynh, Philip A. Roberts, Timothy J. Close

**Affiliations:** ^1^Department of Botany and Plant Sciences, University of California at RiversideRiverside, CA, USA,; ^2^Department of Nematology, University of California at RiversideRiverside, CA, USA

**Keywords:** marker assisted breeding, single nucleotide polymorphism, cowpea, seed size, diversity analysis

## Abstract

Seed size distinguishes most crops from their wild relatives and is an important quality trait for the grain legume cowpea. In order to breed cowpea varieties with larger seeds we introgressed a rare haplotype associated with large seeds at the *Css-1* locus from an African buff seed type cultivar, IT82E-18 (18.5 g/100 seeds), into a blackeye seed type cultivar, CB27 (22 g/100 seed). Four recombinant inbred lines derived from these two parents were chosen for marker-assisted breeding based on SNP genotyping with a goal of stacking large seed haplotypes into a CB27 background. Foreground and background selection were performed during two cycles of backcrossing based on genome-wide SNP markers. The average seed size of introgression lines homozygous for haplotypes associated with large seeds was 28.7g/100 seed and 24.8 g/100 seed for cycles 1 and 2, respectively. One cycle 1 introgression line with desirable seed quality was selfed for two generations to make families with very large seeds (28–35 g/100 seeds). Field-based performance trials helped identify breeding lines that not only have large seeds but are also desirable in terms of yield, maturity, and plant architecture when compared to industry standards. A principal component analysis was used to explore the relationships between the parents relative to a core set of landraces and improved varieties based on high-density SNP data. The geographic distribution of haplotypes at the *Css-1* locus suggest the haplotype associated with large seeds is unique to accessions collected from Southeastern Africa. Therefore this quantitative trait locus has a strong potential to develop larger seeded varieties for other growing regions which is demonstrated in this work using a California pedigree.

## Introduction

Seed size is one of the most universal features that distinguishes domesticated plants from their wild relatives. Larger seeds produce more competitive seedlings under cultivated conditions (Purugganan and Fuller, 2009) and are preferred for most culinary preparations of naked grain. This is true for cowpea (*Vigna unguiculata*) where the demand for large seeds continues for most market classes, especially blackeyes, and rough seed types grown for flour production. Traders, farmers, food vendors, and consumers in West Africa prefer and are willing to pay a price premium for larger cowpea grains (Langyintuo et al., 2004; Mishili et al., 2009; Egbadzor et al., 2013a) so this trait has the potential to improve the income of cowpea growers in regions where ∼125 million people live in poverty (International Fund for Agricultural Development [IFAD], 2001). Cowpea breeders can help meet this demand by developing varieties with larger seeds.

Seed size in cowpea is highly heritable and quantitative, and small seeds are partially dominant to large seeds (Drabo et al., 1984; Egbadzor et al., 2013b,c). Genetic mapping using experimental populations has tagged a few seed size associated quantitative trait loci (QTLs) with markers that could be useful in breeding (Fatokun et al., 1992; Egbadzor et al., 2013b; Lucas et al., 2013a). Interestingly two of these publications report on the orthology of seed size based on comparative mapping to known seed size associated loci in the genomes of cowpea relatives mung bean (*Vigna radiata*) and soy bean (*Glycine max*). Knowledge of marker-trait associations from these studies can be essential components of marker-assisted breeding strategies to help develop varieties with larger seeds.

California Blackeye 27 (CB27) and IT82E-18 are two cultivars that were used to build many of the genomic resources in cowpea. A recombinant inbred population derived from the cross of these two individuals was used to help construct a consensus genetic map of 1,107 expressed sequence tag (EST)-derived SNP loci (Lucas et al., 2011). This population was also used to characterize the inheritance of heat tolerance during reproductive development (Lucas et al., 2013b), resistance to feeding damage caused by foliar thrips (Lucas et al., 2012), and seed size (Lucas et al., 2013a). The *Css*-1 QTL described by Lucas et al. (2013a) provides an attractive breeding opportunity because it is known to be a major determinant of seed size and the haplotype associated with large seeds is absent from the California Blackeye pedigree. In this work we target the introgression of a 4.1 cM *Css-1* haplotype from an African buff seed type variety, IT82E-18, into a California Blackeye variety, CB27 using marker-assisted breeding.

## Materials and Methods

### Principal Component Analysis

The relatedness of CB27 and IT82E-18 was assessed through comparison with a core set of 212 individuals. The majority of the accessions in the core set are landraces which represent the West and South–East African genepools described by Huynh et al. (2013). To understand how CB27 compares, the core set also included other improved varieties from California and landraces representative of other geographic regions including Asia, Europe, the Middle-East, and North Africa. Genotype data for these samples were obtained from Lucas et al. (2011) and Huynh et al. (2013) which utilized the 1,536-plex EST-derived SNP genotyping platform of cowpea (Muchero et al., 2009). A principal component analysis was performed after filtering SNPs for MAF (minor allele frequency) > 0.01 and imputing missing genotype data using the software TASSEL (Bradbury et al., 2007). The first two principal components were plotted on a scatter plot and samples were colorized based on their geographic origin.

### Distribution of *Css-1* Haplotypes

The *Css-1* locus comprises a 4.1 cM region on linkage group 5 of the cowpea consensus genetic map defined by SNP markers 1_0099, 1_0935, 1_0974, and 1_0078 (Lucas et al., 2013a). The haplotype of cowpea variety IT82E-18 characterized by the aforementioned SNPs is associated with the inheritance of large seeds and the genotype calls are GG, GG, GG, and AA, respectively, in Illumina Top Strand format. This unique combination of genotype calls was queried against the diversity panel used in the principal component analysis and the results were tallied by country and by geographic region which helped understand the origin and distribution of the QTL in domesticated cowpea germplasm.

### Introgression of the IT82E-18 *Css-1* Haplotype

Donor parents for both cycles of the marker-assisted breeding project were chosen based on four criteria: (1) Foreground selection for the haplotype associated with large seeds at *Css-1*, *Css-2*, and *Css-4* (Lucas et al., 2013a); (2) Background selection for similarity to CB27 based on 1,536 SNP genotype data; (3) 100 seed weight (Lucas et al., 2013a); and (4) Seed coat type. Using these criteria recombinant inbred line (RILs) -62, -74, -90, and -113 were chosen as donor parents for cycle 1. These lines were crossed to one female CB27 plant to produce four types of F1s. One F1 plant of each type was selfed to produce four F2 families. After 2 weeks of growth, tissue was taken from 45 F2s using the laboratory of the government chemist (LGC) genomics tissue collection method. DNA extraction and genotyping for 49 SNPs was performed using the KASP technology of LGC Genomics. Four of these SNPs distinguish the *Css-1* haplotypes while the others were chosen based on their distribution in the consensus genetic map (Lucas et al., 2011), their linkage to two other QTL affecting seed size that are segregating in this pedigree (Lucas et al., 2013a), and polymorphism between the parents. The genotype calls were compared to *Css-1* haplotypes and to CB27 to generate a ranking of F2s. This analysis was completed before the F2 families finished flowering so another cycle of backcrossing could be immediately pursued. One cycle 1 F2 plant was chosen for additional trials and seed increase because it was homozygous for large seed alleles at *Css-1*, had very large seeds, and a blackeye seed coat. Cycle 2 began by crossing one F2 plant from cycle 1, -113 family, to a female CB27 while another F2 plant from cycle 1, -74 family, was crossed as to CB27 as both a male and a female. The cycle 2 F1s that were produced from these crosses were selfed and 93 F2s comprised of three families (CB27 × Cycle1 -113 F2, CB27 × Cycle 1 -74 F2, and Cycle 1 -74 F2 × CB27) were grown. Tissue from the cycle 2 F2s was collected using the LGC genomics collection method and the DNA was genotyped for 108 SNPs, four of which distinguish the *Css-1* haplotypes. A total of 19 of these SNPs were chosen to assess the content of heat tolerance associated QTLs (*Cht-1*, *Cht-4*, and *Cht-5*) that were characterized by Lucas et al. (2013b). Favorable alleles for heat tolerance QTL *Cht-2* and *Cht-3* are fixed in the cycle 1 parents so markers tagging these loci were not included for additional genotyping. All plants in this work were grown in temperature, irrigation, and pesticide controlled greenhouses. CB27 and IT82E-18 plants were included during each generation as a reference to a known seed size. Seeds were harvested from dried plants, counted, and weighed for each generation to determine the mass of 100 seeds (seed size). The seed type of each plant was observed and categorized as blackeye, browneye, or other. A single factor analysis of variance was performed to test if differences in seed sizes of the RIL population was due to QTL content and QTL stacking.

### Performance Testing

To assess the impact of *Css-1* introgression on other agronomic factors parents and breeding lines were planted on May 29th, 2014 at the University of California, Division of Agriculture and Natural Resources, Kearney Agricultural Research and Extension Center in Parlier, Parlier, CA, USA. Seventy-five seeds were sewn in each experimental plot which were 22-feet long and separated from other plots by a 3-foot alley and 30 inch centered beds. All plots were treated with Temik as a precaution against insects and were well watered every 10 days using furrow irrigation. The experiment was surrounded by buffer plots of the industry standard, CB46. Other California Blackeye cultivars including CB46, CB27, and CB50 in addition to the donor parent from Mozambique, IT82E-18, were grown in blocks of four adjacent plots to compare yield and maturity. 26 cycle 1 F4 families derived from RIL-113 that are homozygous for favorable alleles at all three QTL reported to be segregating in this pedigree by Lucas et al. (2013a) were also tested for maturity, plant height, row closure, visual performance, yield, and 100 seed weight. Maturity was assigned based on six maturity groups including Early, Medium Early, Medium, Medium Late, Late, and Photoperiod Sensitive which was determined by inspecting plots during pod filling (August 25th, 2014). Plant height, row closure, and visual performance were estimated using a number scale of 1–10 by inspecting plots following pod set (August 7th, 2014). Yield was assessed by harvesting all pods in a 3-foot section in the middle of plots (September 25th, 2014). Photoperiod sensitive varieties which grew vegetative and covered neighboring plots prevented yield data collection for eight of the cycle 1 F4 families. These were omitted because they matured and were later over-grown by neighboring plots which prevented sufficient yield sampling (some pods may have been missed due to over growth). One hundred representative seeds were weighed to determine 100-seed weight.

## Results

### Principal Component Analysis

**FIGURE 1:**
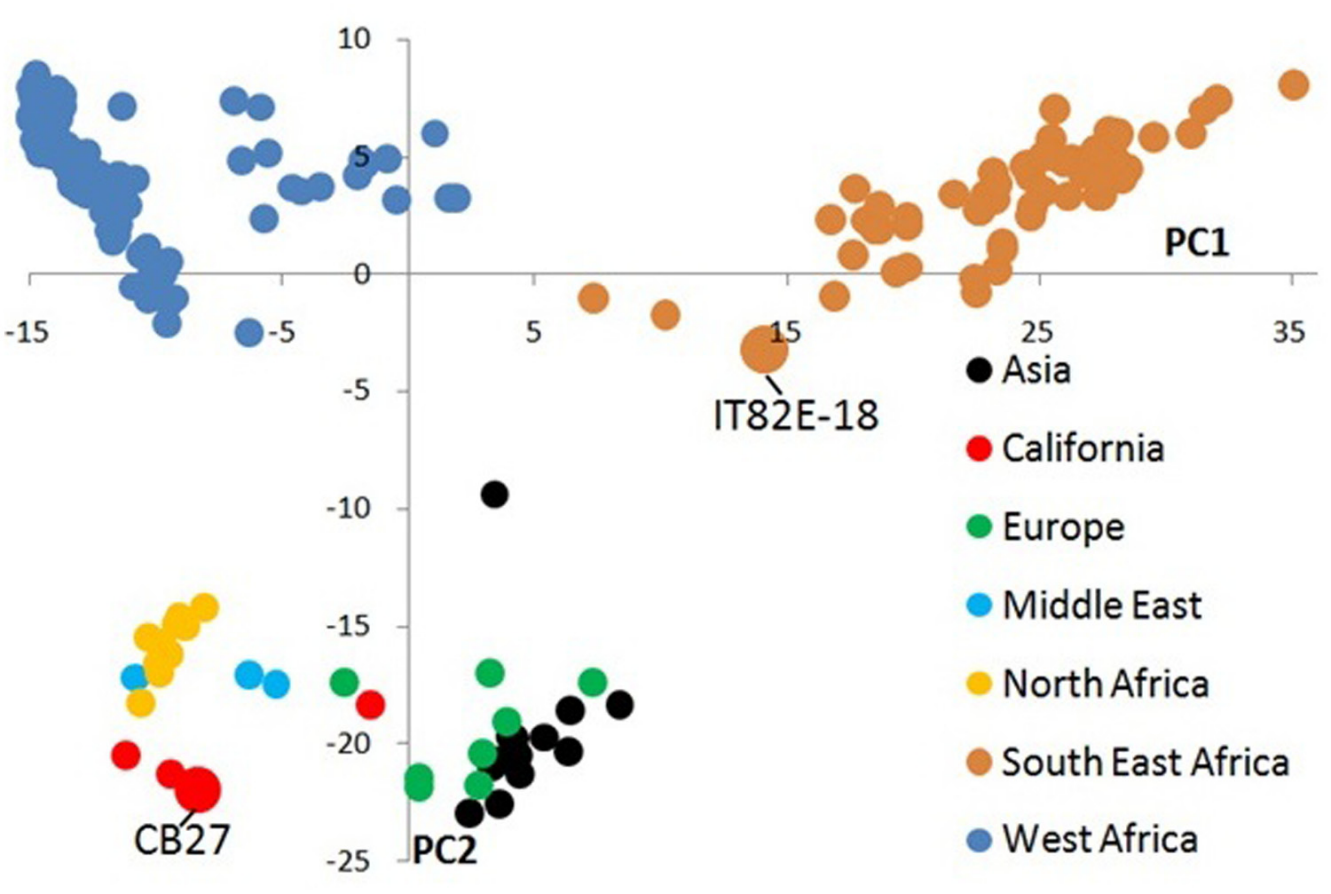
Principal component analysis of 1,536 SNP data from 214 landraces and improved cultivars of cowpea. Samples are colorized based on their geographic origin and the two parents used in this study are labeled. Principle component 1 distinguishes cowpeas collected in West Africa from those collected in South or East Africa. Principle component 2 separates cowpeas primarily collected from outside Africa from those collected within Africa.

A total of 1,073 out of 1,536 SNP markers have MAF>0.01 for the core set of 214 accessions and were used in the principal component analysis (Supplementary Sheet ‘Genotype Data for Tassel’). The first principal component describes 24% of the variation and distinguishes West African landraces from South–East African landraces (**Figure 1**, Supplementary Sheet ‘Variance Explained by PCs’). The second principal component explains 8% of the variation and distinguishes these two centers of diversity from all other landraces and improved California varieties. In the principal component analysis IT82E-18 clusters with landraces from South–East Africa while CB27 forms a cluster with other California varieties, landraces from the Middle East, and North Africa that are only separated from the West-African landraces by principal component 2 (Supplementary Sheet ‘PCA’).

### Distribution of *Css-1*

Out of all 214 accessions studied in the principal component analysis only 19 carry the SNP haplotype of IT82E-18 and all of these are from countries in South and East Africa (Supplementary Sheet “Distribution of *Css-1*"). These include accessions from Botswana, Lesotho, Malawi, Mauritania, Mozambique, South Africa, Uganda, Zambia, and Zimbabwe. Mauritania is a country located in West Africa. However, the one accession (TVu-467) collected from this region that also contains the large seed haplotype at the *Css-1* locus is clearly a migrant or error in records based on the diversity study of Huynh et al. (2013) which associates a probability of 100% that this accession belongs to the Southeastern genepool (Genepool 2). The haplotype is absent from all other accessions including those from West Africa, North Africa, Europe, Middle-East, Asia, and California.

### Introgression of *Css-1*

**FIGURE 2:**
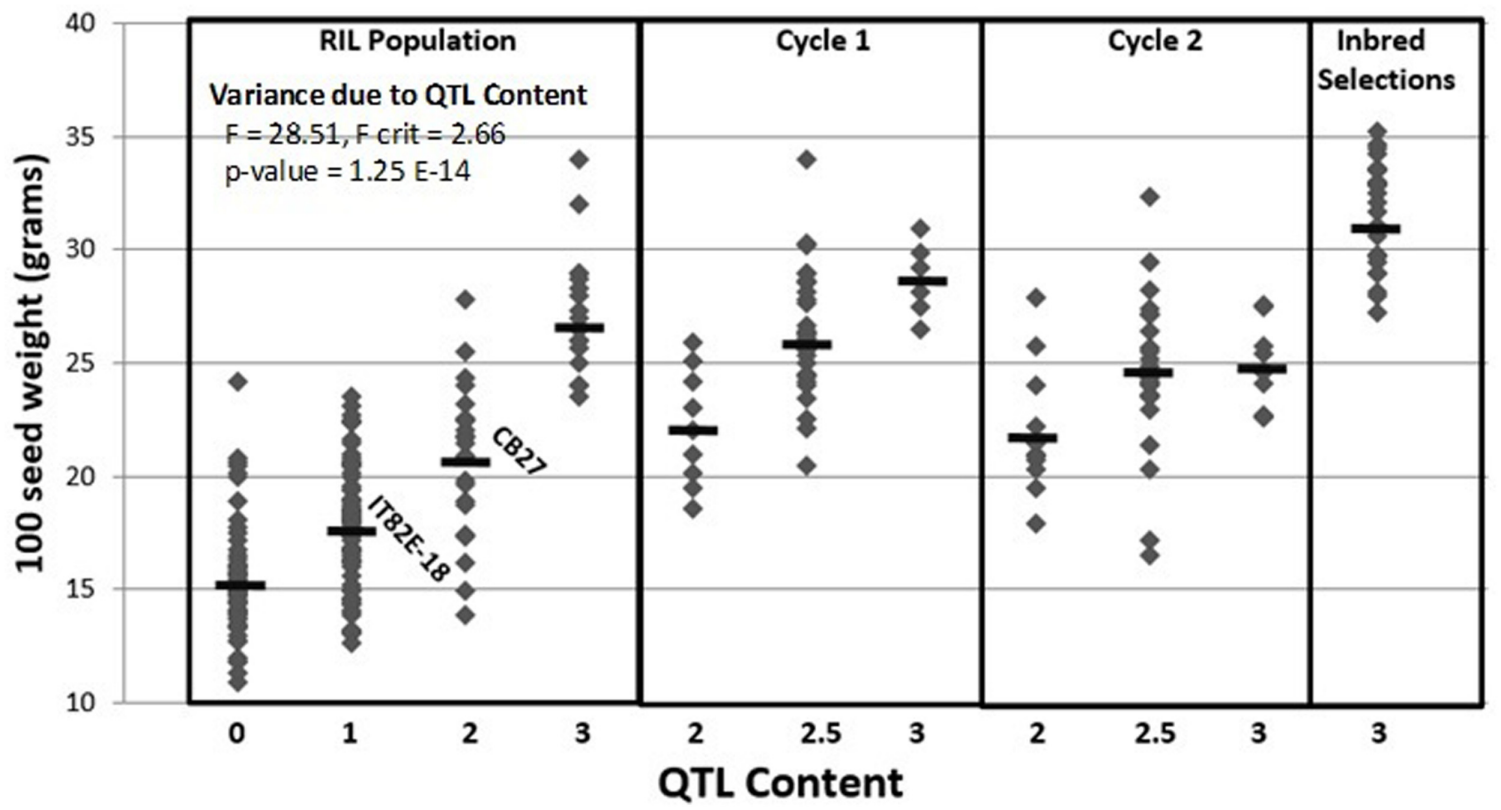
Marker-assisted introgression of the IT82E-18 haplotype at the *Css-1* locus into a CB27 background. Samples are divided into recombinant inbred line population (RIL), cycle 1, cycle 2, and inbred selections. Samples are further divided based on their QTL content for three segregating QTL (i.e., 2.5 means samples are homozygous for large seed alleles at two QTL and heterozygous for large seed alleles at the third QTL).

**FIGURE 3:**
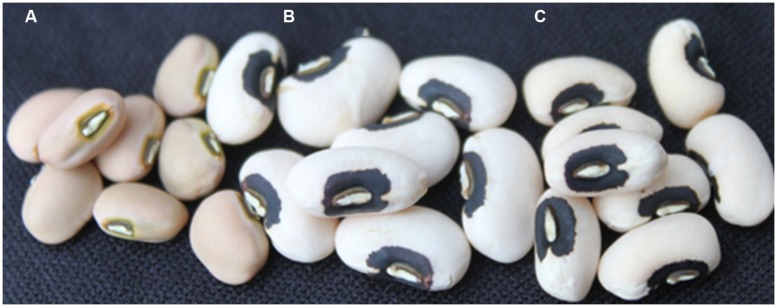
Cowpea seeds for the two parents **(A)** IT82E-18 and **(C)** CB27 used in this work. **(B)** Cycle 1 F4 introgression line homozygous for large seed alleles at all three segregating QTL which has a 100 seed weight of 32 g and a blackeye seed type like the recurrent parent CB27. Each sample has eight seeds represented in the picture.

A total of 435 out of 1,536 SNPs are polymorphic between CB27 and IT82E-18 (Supplementary Sheet ‘Polymorphism of Parents’; Lucas et al., 2011). A genome wide subset of 50 polymorphic SNPs were genotyped for the cycle 1 plants which helped categorize the breeding lines based on foreground and background selection (Supplementary Sheet ‘Cycle 1 Data’). The seed size for cycle 1 plants are plotted in **Figure 2** where homozygotes carrying favorable alleles at *Css-1* had an average seed size of 28.70 g/100 seed while heterozygotes averaged 26.33 g/100 seed and homozygous unfavorable alleles had an average seed size of 22.14 g/100 seed. QTL content has a significant effect on seed size *F*(3,149) = 28.51, *p* = 1.25E-14, ^2^ = 0.36. Only ∼16% of cycle 1 plants produced seeds with the targeted blackeye seed type. One cycle 1 homozygote, -113-2-6, was selfed for two generations and consistently made large seeds which averaged ∼31.68 g/100 seed and is photographed next to the parents in **Figure 3** (Supplementary Sheet ‘Inbred Selection from Cycle 1’). A total of 109 genome wide SNPs were genotyped on the cycle 2 plants (Supplementary Sheet ‘Cycle 2 Data’). Favorable alleles for heat tolerance QTLs *Cht-2*, *Cht-3*, *Cht-4*, and *Cht-5* are primarily fixed among the cycle 2 plants while *Cht-1* is still segregating (Supplementary Sheet ‘Tracking Heat QTL’). The seed size of cycle 2 plants is also plotted in **Figure 2** where homozygotes carrying favorable alleles at *Css-1* had an average seed size of 24.75 g/100 seed, while heterozygotes averaged 24.70 g/100 seed, and homozygous unfavorable alleles had an average seed size of 21.96 g/100 seed. Seed sizes for progeny derived from reciprocal crosses did not significantly differ. About 85% of cycle 2 plants produced the targeted blackeye seed type.

### Performance Testing

In terms of maturity, no introgression lines were as early as CB27 or as late as CB46 because all were categorized in either Medium Early, Medium, or Medium Late maturity groups (Supplementary Sheet ‘Field Trial’). The donor parent IT82E-18 was categorized as Medium Early while the other California Blackeye CB50 matured at the same time as other Medium Late maturity group members.

The introgression lines varied for plant height, row closure, and visual estimate of performance (Supplementary Sheet ‘Field Maturity and Visual’). For these traits CB46 received relatively high scores meaning it was the tallest, had the best row closure, and was visually estimated to perform well. Most introgression lines behaved similar to CB27 and IT82E-18 which are earlier and shorter in plant height although there are a few introgression lines resembling the height and visual performance of CB46 and CB50.

The introgression lines that were tested in the field had much larger seeds than their parents and other industry standards (Supplementary Sheet ‘Field Yield and Size’). Based on the trial of 26 cycle 1 F4 introgression lines had an average 100 seed weight of 32.83 g with the largest family producing a 100 seed weight of 35.06 g. CB50 is the largest California Blackeye and produced seeds with a 100 seed weight of 28.01 g while CB46 is a smaller sized California Blackeye which made seeds with a 100 seed weight of 23.15 g. The two parents of the introgression effort had 100 seed weights of 24.87 and 19.75 g for CB27 and IT82E-18, respectively.

In terms of yield the introgression lines were variable (Supplementary Sheet ‘Field Yield and Size’). Out of all the breeding lines, parents, and industry standards the two highest yielding lines were introgression lines which yielded ∼600 g of naked grain per three foot section. A few introgression lines yielded less than half this much. CB50 yielded the most grain (589.80 g) out of the registered California Blackeye varieties that were tested which outcompeted CB27 (482.7 g), CB46 (488.1 g), and the Mozambican donor of *Css-1*, IT82E-18 (505.58 g).

## Discussion

### Origin and Features of the Parents

CB27 and IT82E-18 represent two very different pedigrees which are grown for different market classes. CB27 produces medium-large blackeye seeds (22 g/100 seed) with a rough seed coat and was bred for production in the San Jaoquin valley of central California, USA. This contrasts with IT82E-18 which is an improved variety released in Mozambique, among other countries, and developed by the IITA in Southeastern Africa which produces medium (18 g/100 seed) light tan seeds that have a smooth seed coat texture. **Figure 3** provides an image of eight seeds of each parent separated by eight seeds of a cycle 1 F4 introgression line. In addition to these morphological and geographic differences these varieties can also be distinguished based on genotype data.

Out of the 13 bi-parental populations genotyped on the GoldenGate platform the population derived from CB27 and IT82E-18 had the second most polymorphic markers (437/∼1200). The principal component analysis in this work also indicates major differences between the parents. CB27 met our expectations by clustering closer to West African varieties than to IT82E-18 because it was developed by breeding California blackeyes with two Nigerian varieties (Ehlers et al., 2000). It was also no surprise to see IT82E-18 localize near the South–East African landraces which is separated from CB27 by both principal components.

### Introgression of IT82E-18 *Css-1* Haplotype

The dramatic differences in seed type and pedigree between the parents used in this study may keep a breeder from wanting to cross the two. Given our goal of increasing the seed size of an already large blackeye, it seems even less intuitive to use a moderate seed size variety like IT82E-18 as a parent. However, our study was informed by an association study which described the potential to breed a larger CB27 by incorporating a 4.1 cM haplotype from IT82E-18 (Lucas et al., 2013a). Furthermore selection based on background markers provided a means to assess and recover all other features of CB27. The outcomes of this marker-assisted breeding project include new breeding lines that have up to 52% larger seeds, the targeted blackeye seed type, and perform well for other traits like yield, maturity and plant architecture under preliminary, early generation field screens.

The work presented in this manuscript does not strictly follow the backcross method because RILs, derived from the recurrent and donor parents, were used as donor parents for *Css-1* introgression and F1s from crosses between RILs and the recurrent parent were selfed and genotyped at the F2 generation rather than making BC1F1s. These RILs were selected as donors because they are homozygous for all of the three QTL segregating in the CB27 × IT82E-18 pedigree and have a greater proportion of recurrent parent genome than the original *Css-1* donor. Genotyping was performed at the F2 rather than the BC1F1 to avoid any impacts of delayed genotyping information (i.e., BC1F1 plants finished flowering before genotype data was produced and interpreted).

None of the three *Css* QTL that are segregating in the CB27 × IT82E-18 pedigree conflict with selection for heat tolerance (*Cht*) QTL described by Lucas et al. (2013b). *Css-2* (donated by the recurrent parent) is distantly linked (44 cM gap) to *Cht-2* (also donated by the recurrent parent) and *Css*-4 (donated by the recurrent parent) is moderately linked (15 cM gap) to *Cht*-3 (also donated by the recurrent parent), therefore the favorable haplotypes for these heat and seed size QTL are in coupling phase and are fixed in the recurrent parent and donor RILs. However, *Cht* – 1, 4, and 5 are tracked in the progeny created in this work because they are not fixed in the donor RILs and are not linked to the segregating *Css* QTL.

This introgression work still requires much more attention if these breeding lines are to be developed into registered varieties. Several important traits need to be assessed including pest resistance and multi-location yield testing. Furthermore, the preliminary field screening of F4 lines should be repeated on inbred materials. Issues for the lines developed in this work also concern seed quality. The introgression lines have very large seeds relative to the diversity found within domesticated cowpea germplasm collections. Their striking size makes them standout visually and they look odd when placed next to other cowpea varieties. When cleaning seeds from harvested pods special attention has to be given to spacing the thresher drums to prevent large grains from splitting. This issue and perhaps fragility of the seed led to a substantial amount of split seeds during processing. Other noticeable features are slight discoloration and easily removable seed coats. This study was not designed to precisely measure maternal or paternal effects, however, based on reciprocal crosses we did not observe evidence to suggest a role for gender in the inheritance of seed size in cowpea. Future work should revisit cycle 1 and cycle 2 materials to advance lines that have non-splitting seeds and seed coats that are not discolored and do not crack because these traits are fixed among the introgression lines that were tested in the field (-113 family). However, if large seeded varieties are needed for culinary preparations where seed coats need to be removed (i.e., akara and moin moin) then the introgression lines from the -113 family could be desirable. While these preparations are most popular in West Africa these large seeded lines that lose their seed coat easily could be useful as a value-added supplement in processed foods in which cowpea flour is incorporated with other flour mixtures to enhance the nutritional profile.

### Performance Trials

Seed size data collected from the field agrees with what was observed in greenhouse experiments. This was not a surprise because seed size is known to be one of the most highly heritable traits which is particularly true for our work in well-watered and pest controlled environments. The most important trait to consider when breeding grain legumes is yield. This study only reports a single season and single site of field based testing on one of several introgression families. The environment behaved expectedly and the trial was a success, however, we designed the experiment as a preliminary trial that was incorporated with planting a larger field experiment. This field trial was primarily conducted to assess variation in seed size and other traits including maturity, plant architecture, and yield.

### SNP Genotyping in Cowpea

One immediate impact of SNP genotyping is the ability to validate crossing records. This has been a particular valuable tool for identifying rogue lines (Lucas et al., 2013c), which is applicable to this work. We noticed from genotype data that one cycle 2 family, 11327, may have arisen from a selfing event because all of the alleles for each line were contributed by CB27. Without genotype knowledge this error may have gone undetected. Future crosses and seed stocks can be validated using SNP genotyping to eliminate lines deviating from a designed pedigree.

### Future Efforts

The *Css-1* haplotype associated with large seeds is unique to cowpea varieties originating from South and East Africa which means it is absent from the West African genepool and also has not been incorporated into varieties for other cowpea growing regions including the Americas and Asia. The distribution of this haplotype in cowpea diversity and its dramatic effect on seed size may be interesting to continue to study because it could relate to domestication. This work builds upon knowledge of the effect of *Css-1* in one pedigree and should not be considered a diagnostic marker that can predict seed size in a random population, which could be explored through future introgression efforts. The potential to combine QTL based selections for different traits is further enhanced when using common genotyping platforms. For example in a recent study of aphid resistance, Huynh et al. (2015) also used the genotyping platform of Muchero et al. (2009) to develop trait-associated markers which could provide a common genotyping resource in attempts to combine aphid resistance and seed size.

There are few warm season legumes that have a substantial amount of genomic resources and knowledge concerning the inheritance of traits like soybean and common bean. Legumes important for food security in developing nations are only recently realizing improvement through genomics-assisted approaches to plant breeding which capitalize on improved genome sequencing technologies and QTL studies (Bohra et al., 2014). For less intensively studied crops like cowpea knowledge of synteny to well-studied relatives provides opportunities to reconcile knowledge across plants from different genera. Lucas et al. (2013a) found that regions of the genome important for the inheritance of seed size are largely conserved between cowpea and soybean. Since that publication the genome sequence of common bean has been released (Schmutz et al., 2014). Cowpea and common bean shared a last common ancestor ∼8 million years ago and are more closely related to each other than to soybean (Lavin et al., 2005). This provides a framework for understanding the genetic mechanism dictated by the *Css-1* locus and for identifying orthologous factors determining seed size. Initial attempts could reconcile the syntenic location of the common bean genes controlling nitrogen metabolism and cytokinin synthesis which are important seed size factors related to common bean domestication. Unfortunately there seems to be no common bean equivalent of Soybase for soybean that catalogs literature findings into a searchable database and genome network.

Determining nutritional profiles of introgression lines and parents are important future experiments for at least two reasons. Comparison of nutritional profiles would allow us to quantify compositional differences in breeding lines from this work that may or may not be desirable for health or cooking characteristics. This should strongly influence our decision to deploy *Css-1* and change how we breed for seed size. Nutritional profiling could also suggest biochemical pathways that could help reveal the genetic mechanism underlying the *Css-1* locus, perhaps through protein variants or gene regulatory elements.

## Supplementary Material

The Supplementary Material for this article can be found online at: http://www.frontiersin.org/journal/10.3389/fpls.2015.00126/abstract
